# Anomalously Suppressed Thermal Conduction by Electron‐Phonon Coupling in Charge‐Density‐Wave Tantalum Disulfide

**DOI:** 10.1002/advs.201902071

**Published:** 2020-04-23

**Authors:** Huili Liu, Chao Yang, Bin Wei, Lei Jin, Ahmet Alatas, Ayman Said, Sefaattin Tongay, Fan Yang, Ali Javey, Jiawang Hong, Junqiao Wu

**Affiliations:** ^1^ Materials Sciences Division Lawrence Berkeley National Laboratory Berkeley CA 94720 USA; ^2^ Department of Materials Science and Engineering University of California Berkeley CA 94720 USA; ^3^ School of Aerospace Engineering Beijing Institute of Technology Beijing 100081 China; ^4^ Advanced Photon Source Argonne National Laboratory Lemont IL 60439 USA; ^5^ School for Engineering of Matter, Transport, and Energy Arizona State University Tempe AZ 85287 USA; ^6^ Department of Mechanical Engineering Stevens Institute of Technology Hoboken NJ 07030 USA; ^7^ Department of Electrical Engineering and Computer Science University of California Berkeley CA 94720 USA

**Keywords:** charge density waves, electron‐phonon coupling, tantalum disulfide, lattice thermal conductivity

## Abstract

Charge and thermal transport in a crystal is carried by free electrons and phonons (quantized lattice vibration), the two most fundamental quasiparticles. Above the Debye temperature of the crystal, phonon‐mediated thermal conductivity (*κ*_L_) is typically limited by mutual scattering of phonons, which results in *κ*_L_ decreasing with inverse temperature, whereas free electrons play a negligible role in *κ*_L_. Here, an unusual case in charge‐density‐wave tantalum disulfide (1T‐TaS_2_) is reported, in which *κ*_L_ is limited instead by phonon scattering with free electrons, resulting in a temperature‐independent *κ*_L_. In this system, the conventional phonon–phonon scattering is alleviated by its uniquely structured phonon dispersions, while unusually strong electron‐phonon (e‐ph) coupling arises from its Fermi surface strongly nested at wavevectors in which phonons exhibit Kohn anomalies. The unusual temperature dependence of thermal conduction is found as a consequence of these effects. The finding reveals new physics of thermal conduction, offers a unique platform to probe e‐ph interactions, and provides potential ways to control heat flow in materials with free charge carriers. The temperature‐independent thermal conductivity may also find thermal management application as a special thermal interface material between two systems when the heat conduction between them needs to be maintained at a constant level.

## Graphical abstract

Electron–phonon interaction that normally plays negligible role in lattice thermal conduction of crystalline materials is found to dominantly scatter phonons and limit thermal conduction in a charge‐density‐wave material, 1T‐TaS_2_. This work elucidates the basic transport behavior of the two most fundamental quasi‐particles in a crystal, free electrons and phonons.

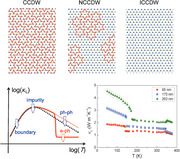

Interactions between free electrons and lattice vibration (phonons) in metallic conductors lead to their electrical conductivity decreasing with temperature, an effect that is widely observed and well understood.^[^ ^1^, ^2^ ^]^ However, effects of electron‐phonon (e‐ph) interaction on materials’ thermal conductivity are not understood as well, and have been experimentally elusive.^[^ ^3^, ^4^ ^]^ In electrical conductors, in addition to directly conducting heat themselves (contributing to thermal conductivity with the electronic part, *κ*_e_), free charge carriers also couple with and scatter phonons, hence reducing *κ*_L_. The reduction in *κ*_L_ arising from e‐ph coupling is typically weak as a very high density (>≈10^20^ cm^−3^) of electrons is needed;^[^ ^4^ ^]^ hence in nonmetallic systems where carrier density is lower, it is hard to be experimentally detected. In metallic systems with very high charge carrier densities, the e‐ph scattering could rise to levels that considerably reduce *κ*_L_, but the measured total thermal conductivity *κ* = *κ*_e_ + *κ*_L_ would then be dominated by the contribution of *κ*_e_ instead. Experimental exploration of the effects of e‐ph scattering on thermal transport has been limited to metallic systems at very low temperatures^[^ ^5^ ^]^ because of the difficulty in separating *κ*_L_ from *κ*_e_.

Indeed, recently Liao et al.^[^ ^4^ ^]^ calculated that *κ*_L_ of silicon can be reduced by up to ≈45% in the presence of a high density (≈10^21^ cm^−3^) of free charge carriers. It has also been proposed that e‐ph coupling may be responsible for unusually low values of *κ* observed in VN*_x_*.^[^ ^6^ ^]^ Yang et al. reported *κ*_L_ reduced in NbSe_3_ nanowires beyond conventional phonon scattering mechanisms that is attributed to e‐ph coupling.^[^ ^7^ ^]^ Theoretical calculations done by Li et al. show that group‐V transition metal carbides (VC, NbC, and TaC) host intrinsically strong e‐ph coupling and weak ph‐ph scattering, leading to *κ*_L_ theoretically much lower than the case when the e‐ph coupling is absent.^[^ ^8^ ^]^ As schematically shown in **Figure** **1**a, we discover direct experimental evidence of *κ*_L_ dominated by e‐ph scattering rather than the conventional ph‐ph scattering, in a charge‐density‐wave material, tantalum disulfide (TaS_2_).

**Figure 1:**
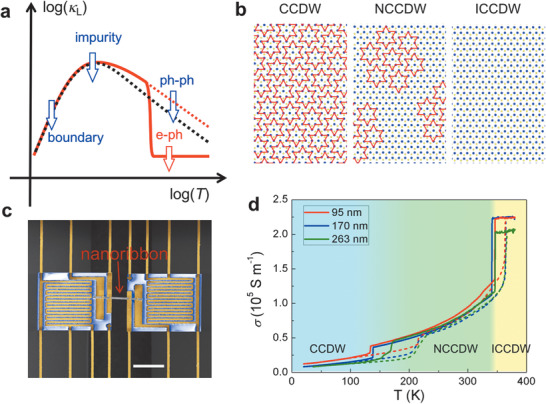
CDW in 1T‐TaS_2_ and nanoribbon devices for electrical and thermal measurements. a) The effect compared to conventional cases: in conventional materials (black dashed line) at high temperatures, lattice thermal conductivity (*κ*_L_) is limited by ph‐ph scattering resulting in an 1/*T* dependence, while the effect of e‐ph scattering is negligible; the opposite is observed in this work, where ph‐ph scattering is intrinsically weak (red dashed line) while e‐ph scattering dominantly limits the thermal conductivity, leading to *T*‐independent *κ*_L_ (solid red line). Major phonon scattering mechanisms at low (boundary) and intermediate (impurity) temperatures are also indicated. b) Schematic charge‐density‐wave structures with different levels of commensuration (commensurate as CCDW, nearly commensurate as NCCDW, incommensurate as ICCDW). Red “David stars” represent displacement patterns of Ta atoms. c) False‐color SEM image of two suspended pads bridged with a TaS_2_ nanoribbon that is FIB‐bonded onto the underlying Pt electrodes. Scale bar: 20 µm. d) Temperature dependence of electrical conductivity of TaS_2_ nanoribbons with different thicknesses measured with a four‐probe geometry. Solid lines are for cooling and dashed lines are for warming.

As a layered material, the octahedral (1T) polytype of TaS_2_ features a well‐known series of charge density wave (CDW) phase transitions.^[^ ^9^, ^10^, ^11^ ^]^ Above ≈550 K, it takes the normal, metallic phase with the space group of *P* $\bar{3}m1$. At lower temperatures, CDW phases show up with distinct commensurations with the underlying lattice: an incommensurate (ICCDW) phase above ≈350 K, a nearly commensurate (NCCDW) phase between ≈150 and ≈350 K, and a commensurate (CCDW) phase below ≈150 K. As schematically shown in Figure 1b, in the CCDW phase, the CDW distortion of atoms forms “David‐stars” resulting in an ordered $\sqrt{13}\times \sqrt{13}$ superlattice structure. The CDW deformation induces a sizeable energy gap in the electronic band structure.^[^ ^12^ ^]^ In the NCCDW phase, domains of David‐star clusters are isolated from each other by a metallic network, where electrons behave itinerant. The CDW phases in 1T‐TaS_2_ are correlated with the lattice deformation and simultaneously a Fermi surface instability,^[^ ^13^ ^]^ where electrons and phonons are strongly coupled to shape its physical properties.

Figure 1c shows the image of a device for electrical and thermal measurements. 1T‐TaS_2_ nanoribbons were fabricated from microflakes mechanically exfoliated out of bulk crystals and measured following the method published previously^[^ ^14^, ^15^ ^]^ (see details in Experimental Section). Electrical conductivity (*σ*) of nanoribbons with different thicknesses is shown in Figure 1d. The three phases (CCDW, NCCDW, and ICCDW) are clearly separated in conductivity at the transition temperature of ≈150 and ≈350 K, respectively, with a hysteresis at each transition. Experimentally, it has been reported in bulk 1T‐TaS_2_ samples that the CCDW phase is a semiconductor and the NCCDW phase is effectively a semimetal,^[^ ^16^ ^]^ and their carrier density is on the order of ≈1 × 10^19^ and ≈1 × 10^22^ cm^−3^, respectively.^[^ ^17^ ^]^

**Figure** **2**a shows measured *κ* over all three phases, qualitatively consistent with that of bulk samples.^[^ ^18^, ^19^ ^]^ In addition to a clear overall reduction of *κ* with nanoribbon thickness, abrupt jumps at each of the phase transition temperature are evident. To extract *κ*_L_, we calculate *κ*_e_ using Wiedemann–Franz law (*κ*_e_ = *LσT*), where the Lorenz ratio *L* takes the Sommerfeld value of *L*_0_ ≡ 2.44 × 10^−8^ W Ω K^−2^, and subtract *κ*_e_ out of the measured *κ*. The obtained *κ*_L_ is shown in Figure 2b. A significant reduction in *κ*_L_ between the CCDW and NCCDW phases is observed at the phase transition temperature around 150 K. More interestingly, in the entire NCCDW phase between ≈150 and ≈350 K, *κ*_L_ becomes nearly *T*‐independent. Since the Debye temperature of 1T‐TaS_2_ is about 172 K,^[^ ^20^ ^]^ in the NCCDW temperature range, *κ*_L_ is expected to be limited by momentum‐nonconserving ph‐ph scattering (the Umklapp process), which would result in an 1/*T* dependence as depicted in Figure 1a (black dashed line). The *T* independence of *κ*_L_, therefore, suggests unconventional phonon scattering mechanisms dominating the Umklapp process in this material.

**Figure 2:**
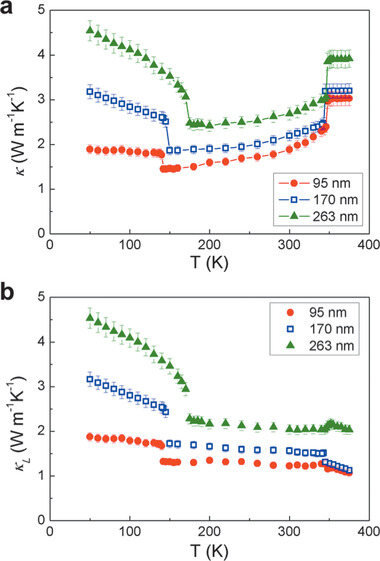
Measured thermal conductivity of TaS_2_ nanoribbons. a) *T* dependence of total thermal conductivity (*κ*) of nanoribbons with different thicknesses. b) Lattice thermal conductivity (*κ*_L_) calculated by subtracting the electron contribution (*κ*_e_) from *κ* assuming the Wiedemann–Franz law.

To suppress the ph‐ph scattering, specific phonon dispersions are required to limit the scattering phase space.^[^ ^8^ ^]^ Due to the lack of long‐range order of the lattice structure in the NCCDW and ICCDW phases, we examine phonon dispersions of the normal phase from the first‐principles calculation (see details in Experimental Section), to provide insights to the NCCDW phase under investigation. Indeed, recent results from angular‐resolved photoelectron spectroscopy shows electronic band structure of NCCDW phase largely in agreement with first‐principles calculations based on the normal phase.^[^ ^16^ ^]^ As shown later, the phonon dispersions we calculated for the normal phase (**Figure** **3**a) are also in good agreement with inelastic X‐ray scattering (IXS) measurements of the NCCDW phase. Two general features are clearly seen in Figure 3a: (1) the acoustic‐optical (a‐o) phonon gap is unusually wide, nearly equal to the bandwidth of the acoustic phonons, similar to the case of BAs^[^ ^21^, ^22^, ^23^, ^24^ ^]^ and NbC;^[^ ^8^ ^]^ and (2) the acoustic phonon dispersions are tightly bunched together. The effect (1) of wide phonon gap is attributed to the large mass ratio in TaS_2_ (*m*_Ta_/*m*_S_ ≈ 5.7),^[^ ^25^ ^]^ as Ta and S are responsible mostly for the acoustic and optical modes, respectively (Figure 3a). Such an a‐o bandgap wider than the maximal acoustic phonon energy prohibits the phonon scattering process that involves two acoustic and one optical phonons (the so‐called *aao* process).^[^ ^21^ ^]^ The effect (2) of acoustic phonon bunching significantly limits the phonon scattering process involving three acoustic phonons (the *aaa* process) by reducing the scattering phase space.^[^ ^21^ ^]^ As a result, the Umklapp process of ph‐ph scattering is greatly suppressed, making room for thermal phonons to be dominantly scattered by other *T*‐independent mechanisms.

**Figure 3:**
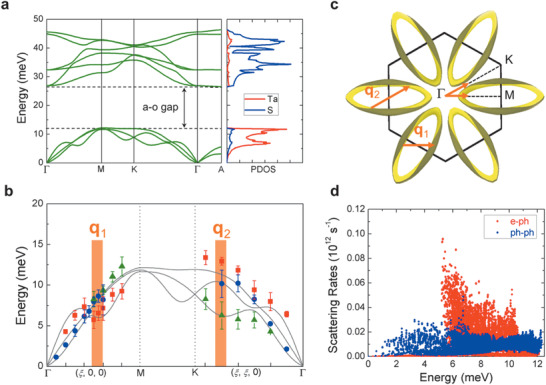
Phonon dispersion and Fermi surface featuring weak ph‐ph scattering and strong e‐ph coupling. a) Phonon dispersion of the normal phase from DFT calculations, and partial PDOS of 1T‐TaS_2_ involving vibration of Ta and S atoms, respectively. The large a‐o phonon gap and bunched acoustic phonons suppress ph‐ph scattering. A high PDOS peak is seen between 6 and 8 meV, corresponding to the less‐dispersive phonon modes. b) Acoustic phonon dispersion (points) of the NCCDW phase measured at 300 K by IXS, overlaid onto the calculated dispersion (solid lines) in (a). The measured phonon linewidth is represented by the error bars. The phonon anomalies along the Γ‐M and Γ‐K directions are clearly seen, where the linewidth is unusually broadened. c) Calculated electron Fermi surface of 1T‐TaS_2_ in the normal phase, which is nested with the two wavevectors ***q***_1_ and ***q***_2_ along the Γ‐M and Γ‐K directions, respectively. These two wavevectors are correlated to the phonon anomalies shown in (b), enabling strong e‐ph coupling. d) Calculated e‐ph (red points) and ph‐ph (blue points) scattering rates as a function of phonon energy, showing e‐ph scattering rates higher than ph‐ph rates for phonon modes between ≈6 and 8 meV.

To understand phonon properties in the NCCDW phase, we measured the acoustic phonon dispersions using IXS (see details in Experimental Section). The phonon dispersions measured at 300 K along Γ‐M and Γ‐K directions are shown in Figure 3b with the underlying calculated dispersions (solid line). It can be seen that these transverse acoustic (TA) and longitudinal acoustic (LA) phonon dispersions are indeed bunched together as shown from density functional theory (DFT) calculation. Secondly, Kohn anomalies^[^ ^26^ ^]^ are observed in the dispersions. For example, along the Γ‐M direction, the LA phonon shows phonon softening at wavevector ***q***_1_ ≈ 0.6 ***q***_Γ‐M_, consistent with neutron scattering measurements.^[^ ^27^ ^]^ Along the Γ‐K direction, the TA phonon modes also show broad, flat regions at certain range of wavevectors denoted as ***q***_2_. Interestingly, these less‐dispersive modes generally show larger linewidths (*Γ*) than dispersive modes at comparable energies, as shown in Figure S5, Supporting Information. Since the phonon linewidth is related to phonon scattering rate (1/*τ*) via the uncertainty principle, *Γ* ∝ 1*/τ*, the experimental data in Figure 3b indicate that the acoustic phonons are unusually strongly scattered near those wavevectors.

To elucidate the mechanism of the momentum‐selective phonon scattering, we calculated the Fermi surface of electrons in TaS_2_, which is shown in Figure 3c and Figure S6, Supporting Information. On the Fermi surface, there exist regions with large areas that are parallel to each other; hence, it is strongly nested with certain fixed wavevectors ***q***. The nesting allows free electrons to be strongly scattered on the Fermi surface if a momentum ℏ***q*** is externally provided by phonons. Interestingly, the shape of the Fermi surface in Figure 3c features two nesting wavevectors along the Γ‐M and Γ‐K directions, respectively, quantitatively consistent with the anomaly wavevectors (***q***_1_ and ***q***_2_) observed in the phonon dispersions in Figure 3b. This agreement is a proof of strong coupling between free electrons and acoustic phonons. The e‐ph scattering rate for the phonon mode *j* with wavevector ***q*** is given by^[^ ^1^, ^8^ ^]^

(1) $$\begin{array}{ccc} \frac{1}{\tau_{e-\mathrm{ph}}} & \propto & \sum_{mn}\int dk{\left|g_{nm,j}^{e-\mathrm{ph}}\left(k,q\right)\right|}^{2}\left[f_{0}\left(\varepsilon_{nk}\right)-f_{0}\left(\varepsilon_{mk+q}\right)\right]\\ & & \times \;\delta \left(\varepsilon_{mk+q}-\varepsilon_{nk}-\hbar \omega_{jq}\right) \end{array}$$

where ε_*n**k***_ and ε_*m**k*** + ***q***_ are electron energies in the initial (*n*, ***k***) and the final (*m*, ***k***+***q***) states, ℏ*ω*_*j**q***_ is energy of the phonon in the state (*j*, ***q***), *f*_0_ is Fermi distribution, $g_{nm,j}^{e-\mathrm{ph}}\left(k,q\right)$ is e‐ph scattering matrix element, and the sums are over the electron bands *n* and *m*. When the Fermi surface is nested with a wavevector ***q***, phonon modes with momentum ℏ***q*** and energy ℏ*ω*_*j**q***_ will be strongly scattered due to the large phase space available in the integration. This is the case for phonon modes near the Kohn anomaly wavevectors ***q***_1_ and ***q***_2_, which are relatively less dispersive and contribute a high phonon density of states (PDOS) between ≈6 and 8 meV (Figure 3a). Acoustic phonon modes at other wavevectors, especially those with smaller wavevectors, experience much weaker scattering rates. This is experimentally evidenced from the very narrow linewidths for phonon modes near the Γ point observed in IXS experiments (Figure 3b). Indeed, as shown in Figure 3d, first‐principles calculations confirm that the e‐ph scattering rates are considerably stronger than ph‐ph scattering rates for acoustic phonon modes of energy higher than 5 meV, especially those between 6 and 8 meV, corresponding to the less dispersive modes near ***q***_1_ and ***q***_2_.

The unusual temperature‐independent thermal conductivity results fundamentally from a nested Fermi surface interplaying with uniquely structured phonon dispersions. These conditions could be met in metallic compounds with large cation/anion mass ratios and signatures of strong e‐ph coupling such as superconductivity and CDWs. As high density of charge carriers can be introduced by a gate field via either electrostatics or field‐induced insulator to metal transition,^[^ ^11^, ^28^ ^]^ the effect also reveals a potential way to electrically and locally tune thermal conduction of solids for nonlinear thermal devices.^[^ ^29^, ^30^ ^]^ New exotic physics of 1T‐TaS_2_, such as quantum spin liquid state,^[^ ^31^ ^]^ may also need to be invoked as possible additional heat carriers. We also note the complication that in the case of very strong e‐ph coupling, a complete separation between lattice and electronic thermal conductivity may not always be possible.^[^ ^32^ ^]^ We further state that the honeycomb domain walls network of topological excitations in NCCDW phase identified recently^[^ ^33^ ^]^ may provide new phonon modes for heat conduction in 1T‐TaS_2_.

## Experimental Section

##### 1T‐TaS_2_ Nanoribbons Device Fabrication

1T‐TaS_2_ flakes were mechanically exfoliated onto a SiO_2_/Si substrate using polydimethylsiloxane, and then nanoribbons (1–2 µm in width and 25–40 µm in length) were produced using electron‐beam lithography (EBL) followed by reactive ion etching.^[^ ^15^, ^34^ ^]^ The flakes were spin coated by poly(methyl methacrylate) (PMMA, C4‐950, 4000 rpm) and baked at 180 °C for 5 min. The PMMA was patterned with EBL, followed by a developing process using methyl isobutyl ketone/isopropyl alcohol (IPA) = 1:3 for 1 min. The exposed TaS_2_ was etched via reactive ion etching using a mixed gas (90% SF_6_ and 10% O_2_, 60 sccm) for several seconds. After the PMMA removal with acetone and rinsing by isopropyl alcohol, TaS_2_ nanoribbons were obtained. Four Ti/Au electrodes were then deposited onto the nanoribbons. To achieve that, a second EBL process was used to expose the electrode areas on the nanoribbons. The exposed areas were Ar^+^ milled (30–60 s) to remove any oxidized or contaminated layer on the surface, and then coated with Ti (10 nm, deposition rate ≈0.5 Å s^−1^) and Au (70 nm, deposition rate ≈1 Å s^−1^) metals using electron beam evaporation (CHA Solution E‐beam evaporator). A lift‐off process was performed in acetone for ≈5 min with gentle shaking, followed by thorough rinsing with IPA. After that, the selected individual TaS_2_ nanoribbon was manually picked up using a sharp tungsten needle (600 nm tip diameter, Cascade Microtech) in a micromanipulator probe station, and transferred onto an empty, suspended‐pad microdevice, aligning the four Ti/Au metal electrodes of the nanoribbon onto the four Pt electrodes on the suspended pads. A small amount of Pt was then deposited along the edge of the predeposited Ti/Au electrodes, to bond them onto the underlying Pt electrodes using a focused ion beam (FIB) (dual‐beam FEI Quanta). Such two‐step contact formation ensures both good ohmic electrical conduction and negligible thermal resistance of the contacts, while minimizing exposure time to the FIB and the resultant sample damage. Figure S1, Supporting Information, shows schematically the fabrication process and images of a nanoribbon device. The thickness of the nanoribbon was confirmed by atomic force microscopy (AFM) as shown in Figure S1b, Supporting Information. A scanning electron microscopy (SEM) image of the device is shown in Figure 1c. After Pt deposition, electrical quality of the electrodes was verified by a linear *I*–*V* relationship (ohmic contact), as shown in Figure S2a, Supporting Information. The devices were annealed at 373 K for 1 h in vacuum chamber to further improve electrical and thermal contacts at the electrodes.

##### Electrical and Thermal Properties Measurements

The electrical resistance and thermal conductance of nanoribbons were measured using suspended‐pad microdevices. Such suspended nanoribbon configuration not only maximally relieves substrate‐imposed strain and trapped‐charge influence, but also ensures that, unlike typical thin film measurements, the electrical and heat fluxes flow along the same path during the measurements. Two SiN*_x_* pads with Pt electrodes were suspended from the Si substrate by long (≈400 µm) and flexural SiN*_x_* arms. Pt serpentine electrodes were patterned on the pads to serve as microheater and thermometer. Four additional Pt line electrodes were deposited for four‐probe electrical resistance measurements of the nanoribbon sample. Applying a DC current (*I* = 0–15 µA) to the microheater on one pad, the temperature on it (hot pad) was raised by *ΔT*_h_. The heat flowed through the nanoribbon to the other pad (cold pad) and raised its temperature by *ΔT*_s_. The base temperature of the two pads (global temperature) was controlled with an external heater (Lakeshore 335 temperature controller) and cryogenic compressor cooler (HC‐4A, Sumitomo Cryogenics). All the measurements were performed under high vacuum (<10^−6 ^Torr) in the vacuum chamber. An AC current with a small amplitude ≈500 nA and frequency ≈1.1 kHz (199 Hz) was applied to the Pt serpentine electrodes to probe Δ*T*_h_ (Δ*T*_s_) on the hot (cold) pad using lock‐in amplifiers on the basis of the temperature coefficient of resistance of Pt electrodes. The temperature coefficient of resistance of the Pt electrodes at individual temperatures over the temperature range of measurements was calibrated. The thermal conductance of the nanoribbon (*G*) is given by $G=\left(P\times \Delta T_{c}\right)/\left(\Delta T_{h}^{2}-\Delta T_{c}^{2}\right)$,^[^ ^14^, ^35^ ^]^ where *P* is the heating power of a microheater, *P* = *I* ^2^  ×  (*R*_h_ + *R*_arm_), and *R*_h_ and *R*_arm_ are the resistance of Pt electrode on the heating pad and suspended arm, respectively. Electrical resistance of the nanoribbon was measured by the four‐probe method using a Keithley nanovoltmeter (2182A) and precision current source (6220). The dimensions of nanoribbons were determined from both SEM and AFM. Data errors were estimated from errors in size of the nanoribbon and *ΔT*_h_ and *ΔT*_c_ on the pads: ≈8% for thermal conductivity and ≈5% for electrical conductivity.^[^ ^14^, ^15^, ^36^ ^]^ Thermal contact resistance of the devices is negligible as shown in Figure S2b, Supporting Information.

##### Inelastic X‐Ray Scattering Measurements

Phonon dispersions of single crystal 1T‐TaS_2_ were measured using IXS technique. Specifically, phonon dispersion branches along the Γ‐M and Γ‐K directions were measured using the HERIX X‐ray spectrometer at beamline 30‐ID‐C at the Advanced Photon Source using 23.7 keV (*λ* = 0.5226 Å) X‐rays with a focused beam spot size of ≈30 µm.^[^ ^37^ ^]^ The 1T‐TaS_2_ single crystal sample was a large bulk flake with a lateral size of 2–3 mm and thickness of 50–100 µm, as shown in Figure S3, Supporting Information. The thickness was selected to obtain the maximum scattering signal in transmission IXS measurements. The flake was adhered onto a copper rod using thermally conductive epoxy, and the rod was then mounted on an evacuated heating stage for rotation in a vacuum chamber. During the measurement, the counting time was in the range of 30–60 s for each energy scan at a constant ***Q***. The measured energy spectra were fitted using a Gaussian function for the elastic peak and a damped harmonic oscillator function for the phonon peaks.^[^ ^38^, ^39^ ^]^ Figure 3b presents the experimental IXS results, where the phonon energy, *E* = ℏ*ω*, is plotted versus the magnitude of the wavevector (***q***) along the Γ‐M and Γ‐K directions in the reciprocal space. Scattering geometry was used to determine the specific polarization of the atomic vibration for these phonon branches. The scattering vector, ***Q***, is defined by the summation of a Bragg peak position and a small vector as ***Q*** = ***G*** + ***q***, where ***G*** is the reciprocal lattice vector, and ***q*** is a wavevector in the 1st Brillouin zone. Therefore, the phonon branches were measured from combinations of ***Q*** and ***q*** with different Bragg peak positions, and the longitudinal and TA polarizations of phonons were measured separately.^[^ ^40^, ^41^ ^]^ As an example for the phonon branch along Γ‐K direction, the LA phonon dispersion was measured at reciprocal‐lattice points ***Q***_LA_ = (1 + *ξ*, 1 + *ξ*, 0) with *ξ* = 0.05–0.3, and the TA phonon dispersion was measured at ***Q***_TA_ = (*ξ*, *ξ*, 4). ***Q***_LA_ and ***Q***_TA_ are the total scattering vectors, and ***q*** = (*ξ*, *ξ*, 0) is the measured phonon wavevectors. In the schematic reciprocal space with *ξ* = 0.15 as shown in Figure S4a (in Supporting Information), the phonon wavevector of ***q*** = (0.15, 0.15, 0) is parallel to ***Q***_LA_ and approximately perpendicular to ***Q***_TA_, which reveals the longitudinal and transverse atomic vibrations with respect to the phonon propagation direction, respectively. Figure S4b, Supporting Information, shows a plot of the experimental energy scan at ***Q***_TA_ = (0.15, 0.15, 4) of the TA branch. The phonon linewidths were extracted from this line shape function convoluted with experimental resolution function. Figure S5, Supporting Information, illustrates the phonon linewidth for dispersive and less dispersive phonon modes in the NCCDW phase of 1T‐TaS_2_ at 300 K, where the less dispersive phonon modes show more phonon linewidth broadening than the dispersive modes at comparable energies.

##### Simulation Methods

First‐principles calculations were performed in the framework of DFT as implemented in the Quantum Espresso (QE) package^[^ ^42^, ^43^ ^]^ with a plane‐wave cutoff of 55 Ry. The DFT calculations were carried out using norm‐conserving pseudopotentials^[^ ^44^ ^]^ to describe the electron‐ion interaction in the local density approximation of the exchange‐correlation interaction. The van der Waals correction was considered with the method of dispersion correction of DFT (DFT‐D3).^[^ ^45^ ^]^ The fully relaxed lattice constants of *a* = 3.350 Å and *c* = 5.954 Å (experimental values:^[^ ^46^ ^]^ *a* = 3.367 Å, *c* = 5.902 Å) were obtained. The 3D Fermi surface structure was simulated with the WANNIER90 code^[^ ^47^ ^]^ and visualized with the XCrySDen package.^[^ ^48^ ^]^ An electronic smearing of 0.05 Ry was applied in the calculation to stabilize the system and prevent it from undergoing the CDW transition.^[^ ^49^, ^50^ ^]^ The phonon‐electron scattering rates were calculated using the EPW (e‐ph coupling using Wannier functions) code^[^ ^51^, ^52^, ^53^ ^]^ within the QE package^[^ ^42^, ^43^ ^]^ following the procedure described by Li et al.^[^ ^8^ ^]^ The phonon dispersions and e‐ph matrix elements were calculated within density functional perturbation theory as implemented in QE on a coarse 9 × 9 × 5 ***q*** grids and electron band structure on a 18 × 18 × 10 ***k*** grid. The coarse grid quantities were interpolated to fine ***k*** and ***q*** grids of 45 × 45 × 21 within the EPW package. The phonon‐phonon scattering rates were calculated using the D3Q package within the QE.^[^ ^54^, ^55^, ^56^ ^]^

## Conflict of Interest

The authors declare no conflict of interest.

## Author Contributions

H.L. and C.Y. contributed equally to this work. J.W. conceived the project. H.L. and J.W. designed the experiments. H.L. fabricated devices and performed the thermal and electrical measurements. H.L., B.W., A.A., A.S., and J.H. performed the inelastic X‐ray scattering measurements. C.Y. and J.H. performed the theoretical calculation. S.T. grew the single crystal flake of sample. H.L., C.Y., B.W., L.J., F.Y., J.H., and J.W. discussed the data. H.L. and J.W. wrote the draft. All authors contributed to discussing the data and editing the manuscript.

## Supporting information

Supporting Information
