# Supplementary material for: Anomalously Suppressed Thermal Conduction by Electron‐Phonon Coupling in Charge‐Density‐Wave Tantalum Disulfide
Source: Adv Sci (Weinh). 2020 Apr 23;7(11):1902071. doi: 10.1002/advs.201902071 (PMC7284197; doi:10.1002/advs.201902071)
Supplement: Supplementary file 1 — Supporting Information [file ADVS-7-1902071-s001.pdf]

Supporting Information

Anomalously Suppressed Thermal Conduction by Electron-Phonon Coupling in Charge-Density-Wave Tantalum Disulfide

Huili Liu, Chao Yang, Bin Wei, Lei Jin, Ahmet Alatas, Ayman Said, Sefaattin Tongay, Fan Yang, Ali Javey, Jiawang Hong, and Junqiao Wu**

1. Crystal structure and lattice parameters of normal phase 1T-TaS_2

Figure S7 shows the crystal structure of the normal phase of the octahedral (1T) polytype of TaS_2. Table S1 compares the lattice parameters from experimental data in literatures, as well as fully relaxed data in the simulation in this work. The lattice parameters theoretically calculated in this work are consistent with the experimental data in literature.

2. Lattice thermal conductivity of 1T-TaS_2: numerical fitting and discussion

In order to fit to the temperature dependence of lattice thermal conductivity ($\kappa_L(T)$), we use the Born-von Karman dispersion to approximate the acoustic phonon dispersion:[3, 4] $\omega = \omega_0 \sin(\pi q/2q_0)$, where ω_0 and q_0 depend on the mass, stiffness and lattice parameters of the crystal. Here $\omega_0 = 2\upsilon q_0/\pi$, where $q_0 = (6\pi^2\delta)^{1/3}$ is the Debye cutoff wavevector, υ is the acoustic phonon velocity in the long-wave limit, and δ is the number density of primitive unit cell.

We first fit to κ_L in the CCDW phase ($T < \sim 150$ K) using the Matthiessen's rule to combine different scattering mechanisms in the nanoribbons. The total phonon scattering rate is written as $1/\tau = 1/\tau_B + 1/\tau_I + 1/\tau_U$, where the three terms are for boundary (τ_B), impurity (τ_I) and ph-ph scattering (τ_U, Umklapp process), respectively. The frequency dependence of impurity scattering rate is expressed by the Rayleigh scattering model as $1/\tau_I = A\omega^4$, where A is a parameter related to defects concentration.[5] The general form of phonon-phonon

scattering rate is $1/\tau_U = B_1 \omega^2 T \exp(-B_2/T)$, where B_1 and B_2 are parameters related to bonding properties of the material.[6] The boundary scattering rate is expressed by $1/\tau_B = \upsilon/D_{eff}$, where D_{eff} is the effective sample size which is related to the particular shape and cross section area of the nanoribbons.[7] The surface are treated as diffusive. The fitting results are shown in Figure S8. The fitting yields $B_1 = 2.9\times10^{-18}$ s K^{-1} and $B_2 = 76$ K. The fitting yields a defects concentration that depends on the nanoribbon thickness, probably due to different extents of FIB damage and oxidization for nanoribbons with different thicknesses. The parameter A is 1.1×10^{-41} s^3, 1.7×10^{-41} s^3, 3.0×10^{-41} s^3 for 263 nm, 170 nm, 95 nm - thick nanoribbons, respectively. For comparison, in Figure S8 we also show the calculated κ_L of the CCDW phase of bulk TaS_2 by setting $D_{eff} = 1$ mm. Afterwards, an additional e-ph scattering term ($1/\tau_{e\text{-}ph}$) is incorporated into the Matthiessen's rule, to fit to the flat κ_L in the NCCDW phase (~150 K < T < ~350 K). During this procedure, the same set of boundary, impurity scattering and Umklapp parameters are used for the given nanoribbon, as the sample is expected to retain the same geometry, purity and largely the same phonon-phonon scattering between the CCDW and NCCDW phases. Hence the total scattering rate is given by $1/\tau_{tot} = 1/\tau_D + 1/\tau_I + 1/\tau_U + 1/\tau_{e\text{-}ph}$. The additional term is $1/\tau_{e\text{-}ph} = C_1/T^{3/2}\exp(-\upsilon^2 C_2/T)\omega/\upsilon$, where C_1, C_2 are parameters related to the mass density, deformation potential, and carrier concentration at the Fermi level.[8] The parameters for the e-ph scattering were obtained from the fitting as $C_1 = 2.9\times10^4$ m s^{-1} K$^{3/2}$, 3.9×10^4 m s^{-1} K$^{3/2}$, 2.8×10^4 m s^{-1} K$^{3/2}$ for 263 nm, 170 nm, 95 nm - thick nanoribbons, respectively. Therefore, the CCDW phase serves as a good reference to calibrate the ph-ph and e-ph scattering parameters in the NCCDW phase. All of these fitting parameters are tabulated in Table S2. The fitted curves are shown in Figure S8, which appropriately explain the nearly T-independent κ_L.

As discussed in the main text, κ_L is dominantly limited by strong e-ph scattering in the NCCDW phase, whereas ph-ph scattering is relatively weak. To highlight the unusually weak

ph-ph scattering in 1T-TaS_2, we compare it with 1T-HfS_2 which has very similar atomic masses and lattice structure. Unlike TaS_2, however, in HfS_2 the a-o bandgap diminishes, and its acoustic phonon dispersions are much less bunched,[9] providing large phase space for both the *aao* and *aaa* processes. This leads to strong ph-ph scattering and lower κ_L in bulk 1T-HfS_2. There is no experimental data reported on κ_L of 1T-HfS_2. Figure S9 shows the comparison of κ_L of 263nm thick TaS_2 nanoribbon (this work, experimental), bulk TaS_2 (experimental),[10] bulk TaS_2 (simulated, D_{eff} = 1 mm), bulk HfS_2 (calculated),[11] and monolayer HfS_2 (calculated).[12] Bulk TaS_2 in the CCDW phase ($T < \sim 150$ K) and monolayer HfS_2 both show the typical $\kappa_L \sim 1/T$ behavior as expected from dominant ph-ph scattering. However, the fact that the extrapolated κ_L value (black dashed line) of bulk TaS_2 is higher than that of bulk HfS_2 (red point) at 300 K is an indication of weaker ph-ph scattering in TaS_2, considering the higher sound velocity and nearly equal specific heat in HfS_2 (Table S3). Considering that experimental thermal conductivity is typically lower than theoretical value due to impurity and boundary scattering, the real difference in κ_L between TaS_2 and HfS_2 might be even larger. The comparison of parameters between 1T-TaS_2 and 1T-HfS_2 is tabulated in Table S3.

It is intriguing to look at the lattice thermal resistivity ($1/\kappa_L$) of 1T-TaS_2, as shown in Figure S10. The jump in $1/\kappa_L$ between the CCDW and NCCDW phase are nearly constant (~ 0.17 m K W^{-1}) for these nanoribbons. They are independent of the nanoribbon thickness. If the thermal resistivity is approximately written as $1/\kappa_L = 1/\kappa_B + 1/\kappa_I + 1/\kappa_U + 1/\kappa_{e\text{-}ph}$, where each term represents the thermal resistivity arising from boundary, impurity, ph-ph, and e-ph scattering, the jump in $1/\kappa_L$ between the CCDW and NCCDW phases could be mainly attributed to the e-ph scattering emerging in NCCDW phase.

3. Phonon dispersion and phonon linewidth in the ICCDW phase

We also collected data of the phonon dispersion and phonon linewidth in the ICCDW phase at 450 K, as shown in Figure S11 and Supplementary Figure S12, respectively. The phonon dispersion shows very similar acoustic phonons compared to the NCCDW phase. The energy of these phonons is consistent with theoretical prediction based on the normal phase (solid line).

4. Comparison of experimental acoustic phonon branches between inelastic X-ray scattering (IXS) and neutron scattering measurements

We compared the acoustic phonon dispersions measured in this work with neutron scattering measurements.[16] Because of lack of data of full phonon branches in the literature, we show in Figure S13 the acoustic phonon branches along the Γ-M direction. It is clear that the acoustic phonon dispersions in this work are consistent with that in the literature. Both measurements show a dip in the longitudinal acoustic (LA) phonon branch along the Γ-M direction, near the wavevector of approximately 0.6 $\boldsymbol{q}_{\Gamma\text{-M}}$, indicating phonons mode softening as the Kohn anomaly.

References

[1] G. Hagg, N. Schonberg, *Arkiv Kemi* **1954**, *Vol: 7, No. 4*.
[2] Y. Liu, R. Ang, W. J. Lu, W. H. Song, L. J. Li, Y. P. Sun, *Appl. Phys. Lett.* **2013**, *102*, 192602.
[3] N. W. Ashcroft, N. D. Mermin, *Solid State Physics*, Holt, Rinehart and Winston, New York **1976**.
[4] C. Kittel, *Introduction to Solid State Physics*, Wiley, New York **1996**.
[5] B. Abeles, *Phys. Rev.* **1963**, *131*, 1906.
[6] Y. J. Han, P. G. Klemens, *Phys. Rev. B* **1993**, *48*, 6033.
[7] A. K. McCurdy, H. J. Maris, C. Elbaum, *Phys. Rev. B* **1970**, *2*, 4077.
[8] B. Liao, B. Qiu, J. Zhou, S. Huberman, K. Esfarjani, G. Chen, *Phys. Rev. Lett.* **2015**, *114*, 115901.
[9] G. Yumnam, T. Pandey, A. K. Singh, *J. Chem. Phys.* **2015**, *143*, 234704.
[10] M. D. Núñez-Regueiro, J. M. Lopez-Castillo, C. Ayache, *Phys. Rev. Lett.* **1985**, *55*, 1931.
[11] K. F. Garrity, *Phys. Rev. B* **2016**, *94*, 045122.
[12] X. Gu, R. Yang, *Appl. Phys. Lett.* **2014**, *105*, 131903.
[13] J. A. Benda, *Phys. Rev. B* **1974**, *10*, 1409.
[14] H. P. B. Rimmington, A. A. Balchin, *J. Mater. Sci.* **1974**, *9*, 343.
[15] I. Lutsyk, M. Rogala, P. Dabrowski, P. Krukowski, P. J. Kowalczyk, A. Busiakiewicz, D. A. Kowalczyk, E. Lacinska, J. Binder, N. Olszowska, M. Kopciuszynski, K. Szalowski, M.

Gmitra, R. Stepniewski, M. Jalochowski, J. J. Kolodziej, A. Wysmolek, Z. Klusek, *Phys. Rev. B* **2018**, *98*, 195425.
[16] K. R. A. Ziebeck, B. Dorner, W. G. Stirling, R. Schollhorn, *J. Phys. F: Met. Phys.* **1977**, *7*, 1139.

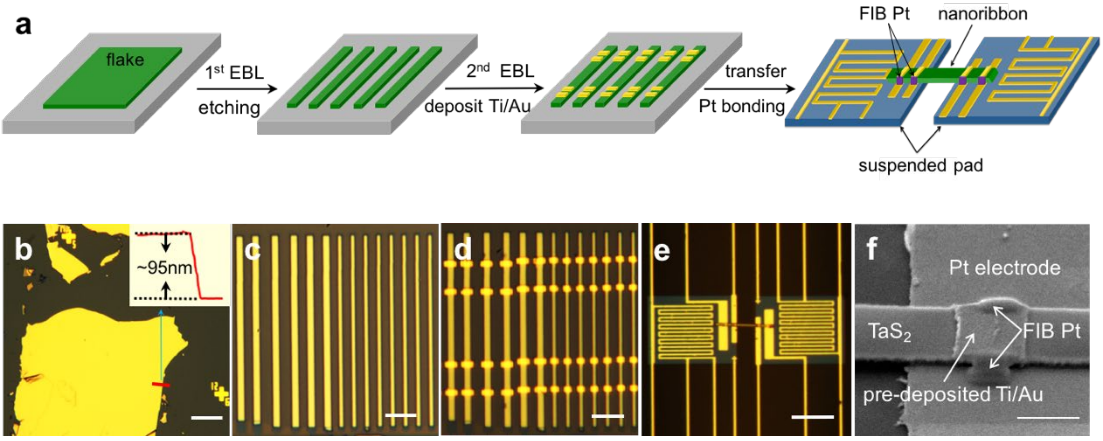

Figure S1. 1T-TaS_2 nanoribbons device fabrication. a) Schematic representation of the TaS_2 nanoribbon fabrication process. Optical images of the flake (b), nanoribbons (c, d), and device (e). Inset in (b) shows the thickness of the flake measured along the red line by an AFM. f) A SEM image of the nanoribbon that was FIB-bonded onto the underlying Pt electrode. Scale bar: 50 µm (b), 5 µm (c, d), 20 µm (e), 1 µm (f).

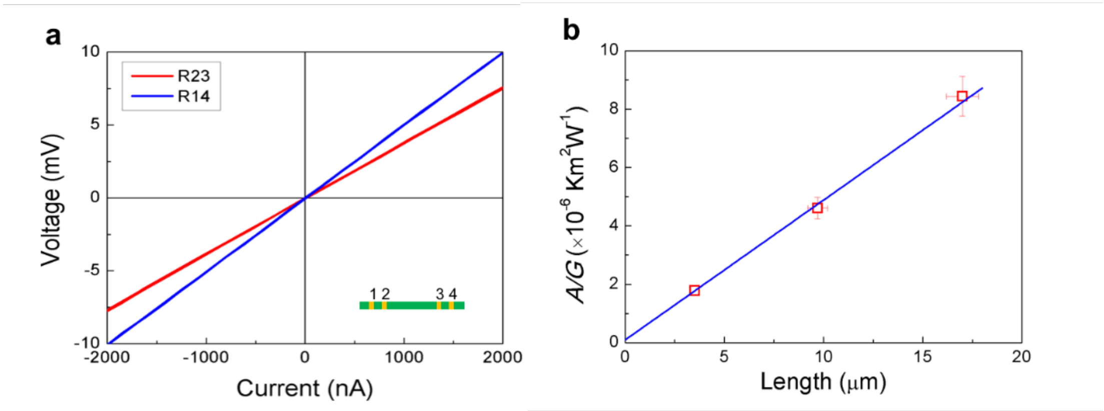

Figure S2. a) Linear I-V relationship of the nanoribbons based on a two-probe measurement, indicating an electrical ohmic contact between the nanoribbon and the electrodes. b) Plot of total thermal resistance (1/*G*) multiplied by cross-sectional area (*A*) as a function of the nanoribbon length (measured at 300K), indicating that the thermal contact resistance between the nanoribbon and pads is negligible. Error bars include errors ~8% for *A*/*G* and ~5% for the nanoribbon length from thermal conductance and dimension measurements of nanoribbons, respectively.

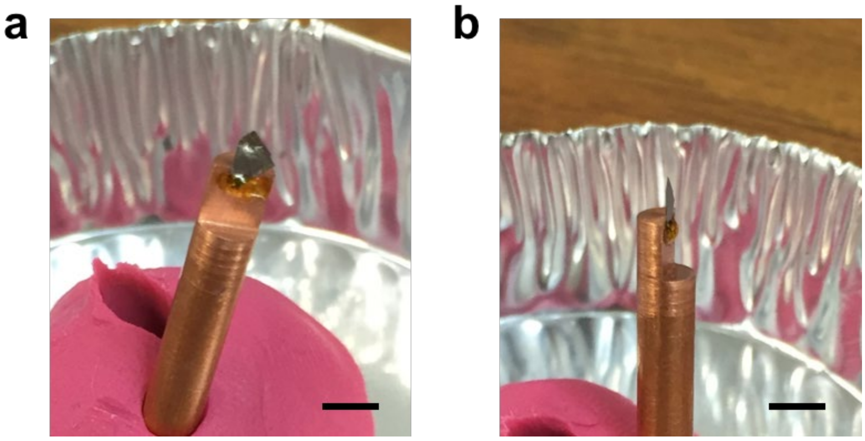

Figure S3. Optical image of a 1T-TaS_2 single crystal flake mounted on the copper rod. The single crystal flake has a lateral size of 2 ~ 3 mm and a thickness of 50 ~ 100 μm for the inelastic X-ray scattering measurements. Scale bar: 5 mm (a), (b).

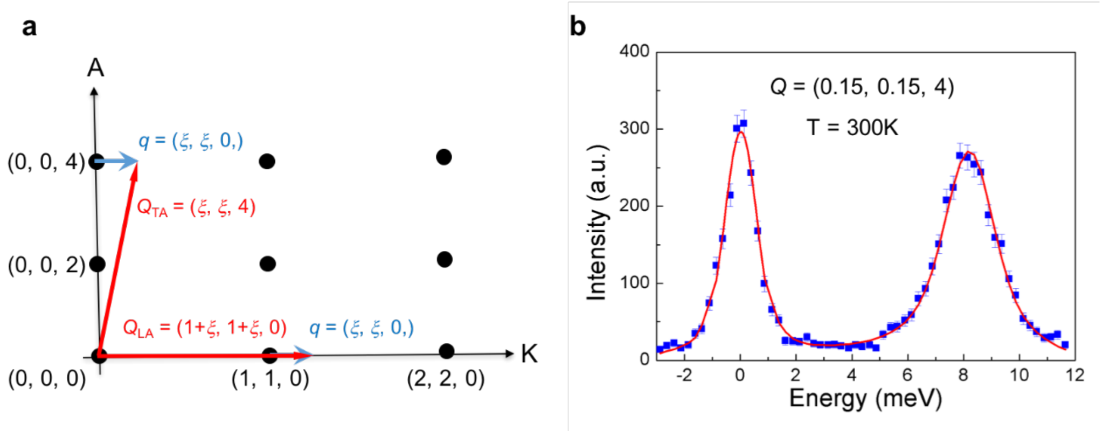

Figure S4. IXS measurements for individual phonon branch. A schematic representation of IXS measurements for individual phonon branches. a) Total scattering vectors, $\boldsymbol{Q}_{LA}$ and $\boldsymbol{Q}_{TA}$, for separate measurements of longitudinal and transverse phonons with wavevector $\boldsymbol{q} = (\xi, \xi, 0)$ along the Γ-K symmetry direction. b) Plot of the experimental energy scan at $\boldsymbol{Q} = (0.15, 0.15, 4)$ in the transverse acoustic branch at 300 K. The line-shape is well fitted by the damped harmonic oscillator model for the inelastic peak and a Gaussian function for the elastic peak.

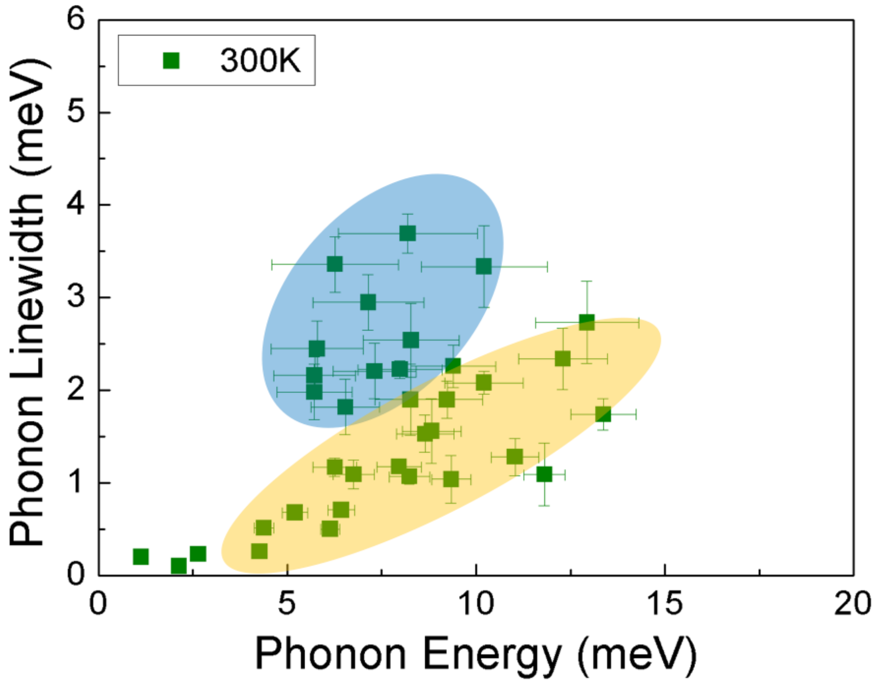

Figure S5. Phonon linewidth versus phonon energy at 300 K. Phonon linewidth for dispersive (yellow ellipse) and less-dispersive (blue ellipse) phonon modes in NCCDW phase. The linewidth was extracted from the Inelastic X-Ray Scattering measurements. For the less-dispersive phonon modes in the range of 5~10 meV, the phonon linewidths are unusually broadened from those of the dispersive modes. In the NCCDW phase of 1T-TaS_2, the lattice vibration is strongly damped at these less-dispersive phonon modes, which is attributed to the strong e-ph interaction mediated by the Fermi surface nesting.

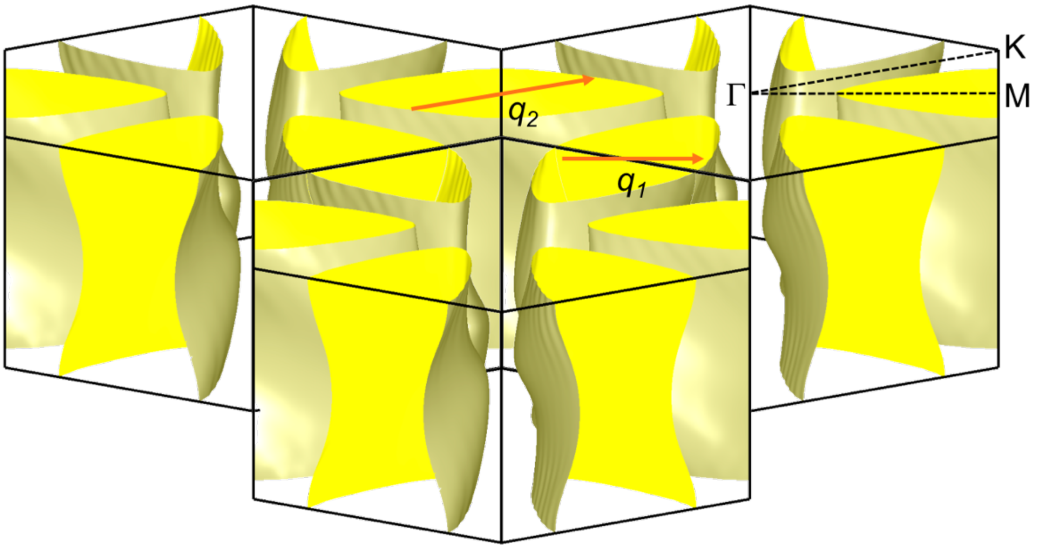

Figure S6. Calculated Fermi surface (3D) of 1T-TaS_2 in the normal phase. This shows, in three-dimensional view, that the Fermi surface is nested with the two electron wavevectors $\boldsymbol{q_1}$, and $\boldsymbol{q_2}$ along the Γ-M and Γ-K directions, respectively.

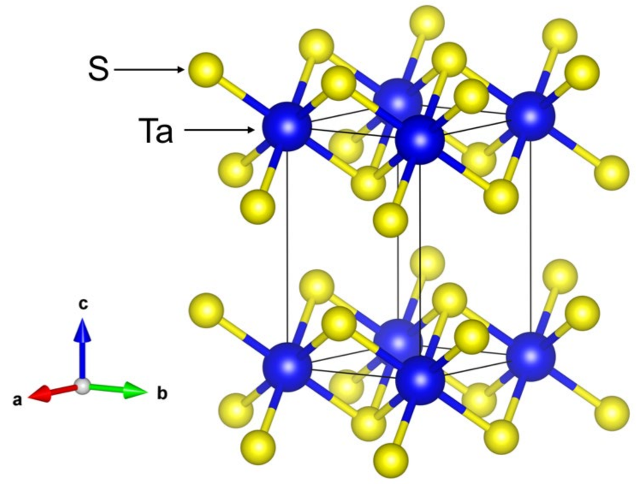

Figure S7. Crystal structure of normal phase 1T-TaS_2.

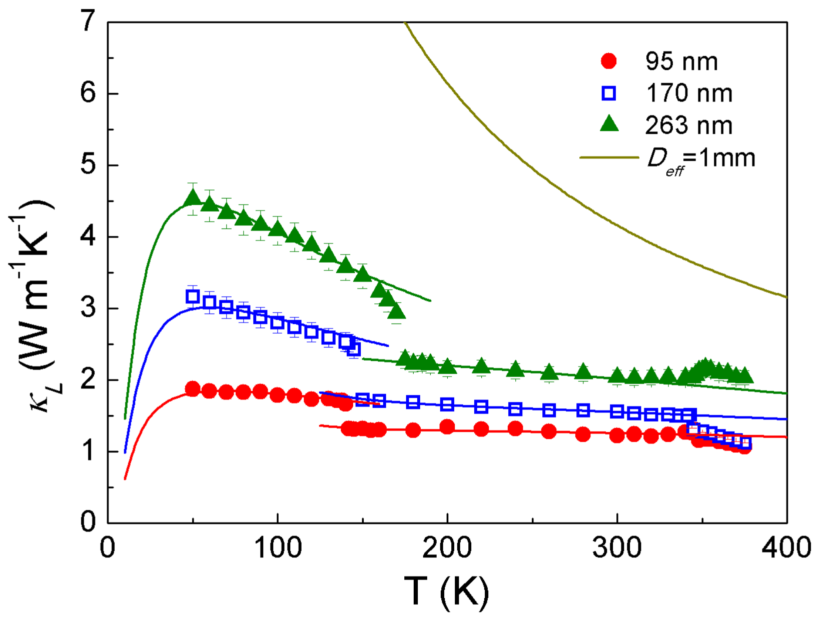

Figure S8. Lattice thermal conductivity (κ_L) obtained by subtracting electron contribution (κ_e) from measured κ assuming the Wiedemann-Franz law ($\kappa_e = L_0\sigma T$). Lines are fitting using total scattering rate of $1/\tau = 1/\tau_B + 1/\tau_I + 1/\tau_U$ for the CCDW phase and $1/\tau = 1/\tau_B + 1/\tau_I + 1/\tau_U + 1/\tau_{e\text{-}ph}$ for the NCCDW phase. As a comparison, we also plotted κ_L simulated for bulk TaS_2 (using D_{eff} = 1 mm), following the simulation model and parameters used for the CCDW phase.

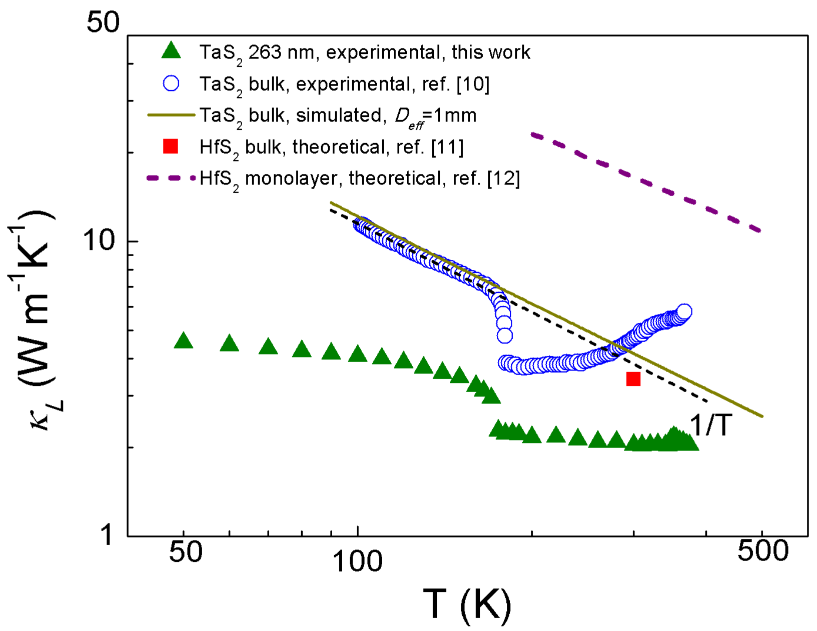

Figure S9. Comparison of lattice thermal conductivity of 1T-TaS_2 and 1T-HfS_2. Black dashed line indicates the typical 1/T-dependence of κ_L in the CCDW phase of bulk TaS_2 limited by phonon-phonon scattering.

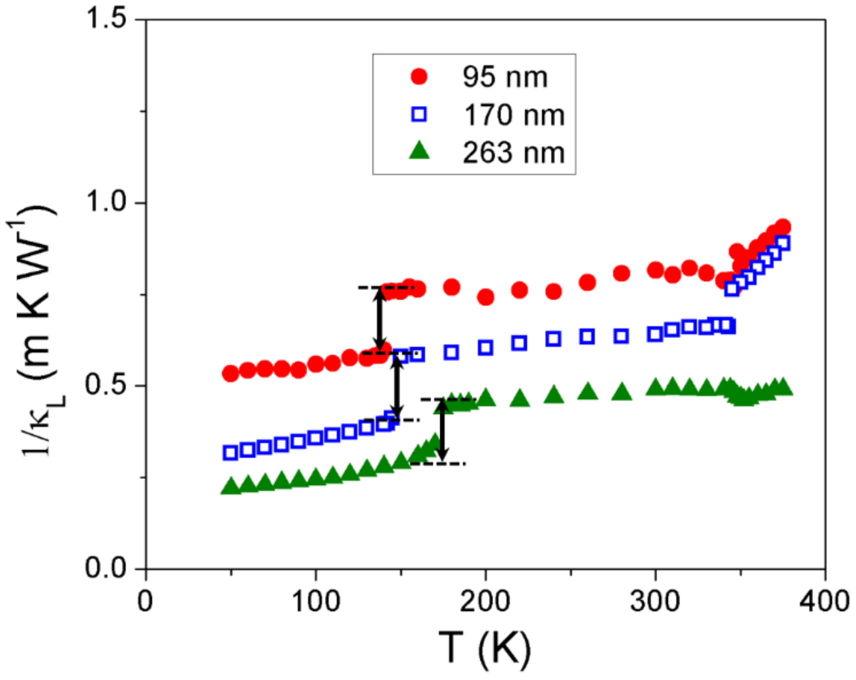

Figure S10. Temperature dependence of the lattice thermal resistivity ($1/\kappa_L$). Nearly the same amount of reduction in $1/\kappa_L$ going from the CCDW to the NCCDW phase was observed for nanoribbons with different thicknesses. The total scattering rate is $1/\tau_{tot} = 1/\tau_0 + 1/\tau_{e\text{-}ph}$, where $1/\tau_0 = 1/\tau_B + 1/\tau_I + 1/\tau_U$. The total lattice thermal resistivity is approximately expressed by

$1/\kappa_L = 1/\kappa_0 + 1/\kappa_{e\text{-}ph}$, hence the amount of jump in $1/\kappa_L$ in this figure is attributed mostly to e-ph coupling, which is ~ 0.17 m-K W^{-1} as indicated by black arrows.

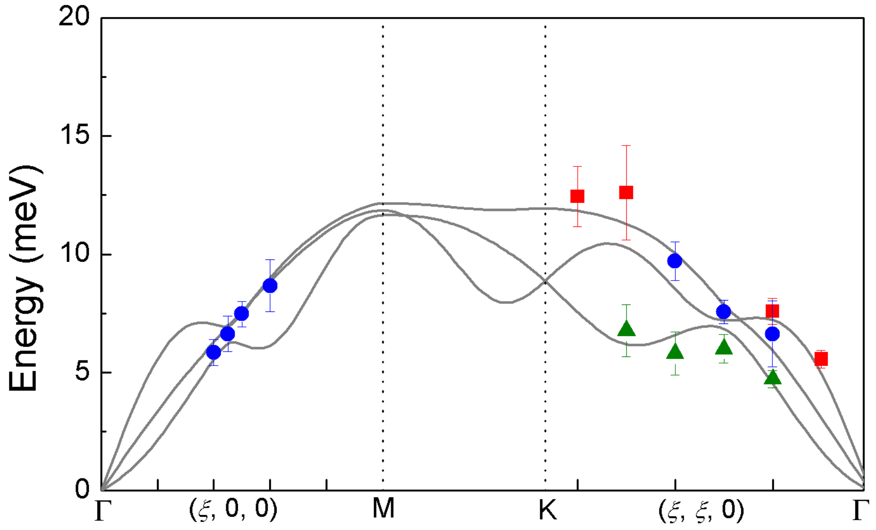

Figure S11. Acoustic phonon dispersion of the ICCDW phase measured at 450K by inelastic X-ray scattering (IXS), overlaid onto the calculated dispersion. The measured phonon linewidth is represented by the error bars.

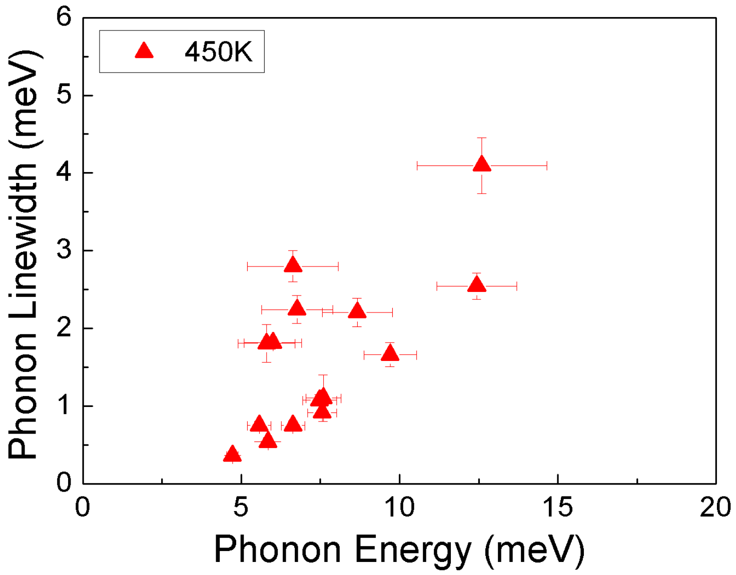

Figure S12. Phonon linewidth versus phonon energy for the ICCDW phase of 1T-TaS_2 at 450K.

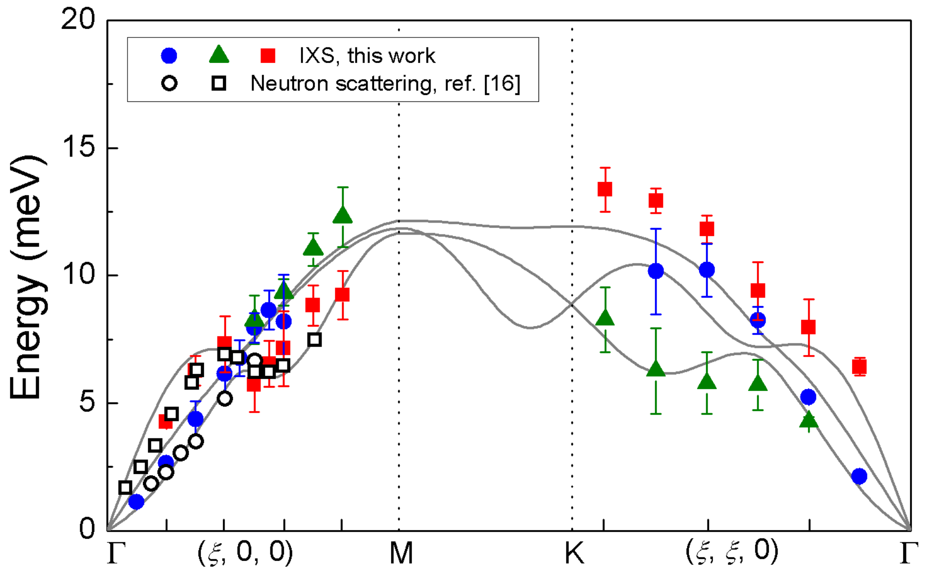

Figure S13. Comparison of phonon dispersions obtained from inelastic X-ray scattering (IXS) and from neutron scattering measurements. Solid points are from IXS measurements, and open points are extracted from the literature (ref. [16]) of neutron scattering measurements.

Table S1. Experimental and calculated lattice parameters of 1T-TaS_2.

	Experimetal[1]	Experimental[2]	Calculated (this work)
Space group		P$\bar{3}$m1 (164)	
Z		1	
a (Å)	3.350	3.367	3.350
b (Å)	3.350	3.367	3.350
c (Å)	5.860	5.902	5.954
α		90^0	
β		90^0	
γ		120^0	
Volume (Å^3)	56.953	57.955	57.867

Table S2. Parameters obtained from κ_L(T) fitting in the CCDW and NCCDW phases of 1T-TaS_2.

	Nanoribbons	Device 1	Device 2	Device 3
	Lattice parameters	$a = b = 3.350$ Å, $c = 5.954$ Å, $\alpha = \beta = 90^0$, $\gamma = 120^0$		
	Debye temperature θ_D (K)	172 [13]		
	Thickness (nm)	95	170	263
	Width (μm)	1.20	1.14	1.34
CCDW	D_{eff} (nm)	268	401	569
	B_1 (s K^{-1})	2.9×10^{-18}		
	B_2 (K)	76		
	A (s^3)	3.0×10^{-41}	1.7×10^{-41}	1.1×10^{-41}
NCCDW	D_{eff} (nm)	268	401	569
	B_1 (s K^{-1})	2.9×10^{-18}		
	B_2 (K)	76		
	A (s^3)	3.0×10^{-41}	1.7×10^{-41}	1.1×10^{-41}
	C_1 (m s^{-1} $K^{3/2}$)	2.8×10^4	3.9×10^4	2.9×10^4
	C_2 (s^2 K m^{-2})	4.2×10^{-5}		

Table S3. Comparison of physical parameters and properties between 1T-TaS_2 and 1T-HfS_2.

	1T-TaS_2	1T-HfS_2
Space group	$P\bar{3}m1$ (164)	$P\bar{3}m1$ (164)
Lattice parameters	$a = b = 3.350$ Å, $c = 5.954$ Å $\alpha = \beta = 90^0$, $\gamma = 120^0$	$a = b = 3.623$ Å, $c = 5.841$ Å $\alpha = \beta = 90^0$, $\gamma = 120^0$ [14]
Atomic masses	Ta/S = 181/32	Hf/S = 178/32

Debye temperature θ_D (K)	172 [13]	289.5 [11]
$\hbar\omega_{min}^{optical}$ (meV)	26.5	16.9 [9]
$\hbar\omega_{max}^{acoustic}$ (meV)	11.6	17.7 [9]
a-o gap ratio, $(\hbar\omega_{min}^{optical} - \hbar\omega_{max}^{acoustic})/\hbar\omega_{max}^{acoustic}$	~ 1.3	~ 0
Specific heat capacity (J mol^{-1} K^{-1}, 300K)	23.6	23.7
Speed of sound (m s^{-1})	2235	3935
κ_L (bulk, 300 K, W m^{-1} K^{-1})	~ 4.7 [10] (experimental)	3.4 [11] (theoretical)
Band gap (eV)	metallic (300 K) [15]	1.6 [11]
